# P05 The impact of COVID-19 on antifungal stewardship in a UK tertiary teaching hospital: a review of prescribing practices and therapeutic drug monitoring of posaconazole

**DOI:** 10.1093/jacamr/dlac053.005

**Published:** 2022-05-31

**Authors:** Adam Andreani, Netta Tyler, Christiane Micallef, David Enoch, Vanessa Wong

**Affiliations:** 1 Department of Haematology, Addenbrooke’s Hospital, Cambridge University Hospitals NHS Foundation Trust, Cambridge, UK; 2 Pharmacy Department, Addenbrooke’s Hospital, Cambridge University Hospitals NHS Foundation Trust, Cambridge, UK; 3 Clinical Microbiology & Public Health Laboratory, UK Health Security Agency, Cambridge, UK; 4 Microbiology Department, Addenbrooke's Hospital, Cambridge University Hospitals NHS Foundation Trust, Cambridge, UK

## Abstract

**Background:**

Posaconazole is a triazole antifungal licensed for the prevention of invasive fungal infections (IFI) in haemato-oncology patients and treatment of IFI where first-line therapy has failed. In January 2020 COVID-19 hit the UK, with many NHS services reduced to meet the unprecedented challenges of the pandemic. We audited prescriptions and therapeutic drug monitoring (TDM) of posaconazole and compared the results with an audit conducted pre-COVID-19 to assess the impact of the pandemic on antifungal stewardship (AFS).

**Methods:**

Inpatients prescribed posaconazole over two 3 month periods (1 October to 31 December 2019 and 2020) at Addenbrooke's Hospital, Cambridge, were identified using an electronic computer system and their records reviewed for demographic, diagnostic and therapeutic data. Posaconazole levels were performed at the national Mycology Reference Laboratory, Bristol, using guide levels of 0.7–3.75 mg/L (prophylaxis) and 1–3.75 mg/L (treatment). The first pre-dose level for each patient was used in analyses. Audit standards included (i) all inpatients on posaconazole to have an indication in local guidelines; (ii) to have the correct loading dose prescribed; (iii) to have a pre-dose level taken (iv) at the correct time (i.e. between Day 3–8) after commencing therapy or a dose change; (v) to have a turnaround time of ≤5 days; and (vi) to stop posaconazole appropriately.

**Results:**

Forty-two 42 inpatients received posaconazole in 2020 (49, 2019). In total, 39/42 (93%) patients had an appropriate indication [46/49, (94%), 2019]: 38/40 (95%) for prophylaxis [45/45, (100%) 2019]; 1/2 (50%) for treatment [1/1, (100%) 2019]. In 2020, 34/42 (81%) of patients received the correct loading dose [41/46 (89%), 2019). TDM was performed on 33/42 (79%) patients [46/46 (100%), 2019], 18/42 (43%) 3–8 days after starting [25/46 (54%), 2019] and 3/13 (23%) after a dose change [9/10 (90%), 2019]; 32/33 (97%) of TDM results were reported in ≤5 days [32/46 (70%), 2019]. Posaconazole was stopped in 24/26 (92%) patients who were no longer receiving immunosuppression and/or neutropenia had resolved [23/23 (100%), 2019].

**Conclusions:**

Despite the pressures of COVID-19, there were good standards of care with only slight reductions in compliance, with the exception of TDM. This may have reflected the change from face-to-face to virtual meetings with haemato-oncology, as well as representing issues of staff shortages and changes in hospital priorities. Such data have highlighted the usefulness of technology to facilitate AFS, e.g. electronic prescribing order sets, which would provide us with quality assurance in a system that will be facing ongoing pressures from the pandemic for years to come.

